# Safe, Effective, and Inexpensive Clearance of *Mycoplasma* Contamination from Cultures of Apicomplexan Parasites with Sparfloxacin

**DOI:** 10.1128/spectrum.03497-22

**Published:** 2022-10-03

**Authors:** Darlene R. Malave-Ramos, Kit Kennedy, Melanie N. Key, Zhicheng Dou, Björn F. C. Kafsack

**Affiliations:** a Department of Microbiology & Immunology, Weill Cornell Medicine, New York, New York, USA; b ACCESS Summer Research Program, Weill Cornell Medicine, New York, New York, USA; c University of Puerto Rico at Mayagüez, Mayagüez, Puerto Rico; d Department of Biological Sciences, Clemson University, Clemson, South Carolina, USA; e Eukaryotic Pathogens Innovation Center, Clemson, South Carolina, USA; University of Georgia

**Keywords:** *Mycoplasma*, contamination, Apicomplexan parasites, antibiotics, sparfloxacin, *Plasmodium*, *Toxoplasma*, *Babesia*

## Abstract

Most commercial products cannot be used for clearance of *Mycoplasma* contamination from cultures of apicomplexan parasites due to the parasites’ dependence on the apicoplast, an essential organelle with DNA replication and translation machinery of cyanobacterial origin. The lone exception, mycoplasma removal agent (MRA), is relatively expensive, and some mycoplasma strains have shown resistance to clearance with MRA. Here, we report that the fluoroquinolone antibiotic sparfloxacin is a safe, effective, and inexpensive alternative for treatment of mycoplasma contamination in cultures of apicomplexan parasites. Sparfloxacin cleared both MRA-sensitive and MRA-resistant mycoplasma species from P. falciparum cultures at 1 and 4 μg/mL, respectively. We show that cultures of three different apicomplexan parasites can be maintained at concentrations of sparfloxacin required to clear mycoplasma without resulting in substantial deleterious effects on parasite growth. We also describe an alternative low-cost, in-house PCR assay for detecting mycoplasma. These findings will be useful to laboratories maintaining apicomplexan parasites *in vitro*, especially in low-resource environments, where the high cost of commercial products creates an economic barrier for detecting and eliminating mycoplasma from culture.

**IMPORTANCE** These findings will be useful to laboratories maintaining apicomplexan parasites *in vitro*, especially in low-resource environments, where the high cost of commercial products creates an economic barrier for detecting and eliminating *Mycoplasma* from culture.

## INTRODUCTION

Contamination with *Mycoplasma* species (class *Mollicutes*) is a common problem in continuous cell culture and found in 15 to 35% of cell lines (1–3), including cocultures of apicomplexan parasites and their host cells. Dozens of mycobacterial species have been found as part of the human microbiota, including skin and hair, but only a small number have been linked to human disease. With a 0.15- to 0.3-μm diameter, Mycoplasmas are some of the smallest known cellular organisms and can pass through the standard 0.2-μm filters typically used in the preparation of culture media. Contamination of cell cultures with *Mycoplasma* is easily missed for several reasons, including their small size, poor staining due to the absence of a cell wall, and failure to change culture turbidity or pH (4). Unlike contamination with most other bacteria or fungi, the presence of *Mycoplasma* rarely results in the collapse of the cell culture but can nonetheless substantially alter the properties and behavior of infected cell lines, making it a potentially important confounder in experimental comparisons.

*Mycoplasma* species are broadly resistant to many classes of commonly used antibiotics, including beta-lactams, glycopeptides, fosfomycin, polymyxins, sulfonamides, rifampicin, narrow-spectrum quinolones, and trimethoprim. Conversely, they remain susceptible to some tetracyclines, macrolides, and quinolones (5). These classes form the basis of commercial products aimed at eradicating *Mycoplasma* contamination from cell cultures, such as Bayer’s Ciprobay and PromoCell’s Biomyc-3 (both the fluoroquinolone ciprofloxacin), PromoCell’s Biomyc-1/2 and Roche’s BM-Cyclin (both are a combination of the macrolide tiamulin and the tetracycline minocycline), InvivoGen’s Plasmocin (a combination of a proprietary fluoroquinolone and a proprietary macrolide), Bayer’s Baytril (the fluoroquinolone enrofloxacin), and MP Biomedicals’ *Mycoplasma* removal agent (MRA; a proprietary fluoroquinolone).

Unfortunately, nearly all these products also inhibit the growth of Plasmodium falciparum (6–8) or other apicomplexan parasites (9, 10) at similar or lower concentrations than those required to effectively clear *Mycoplasma* (11–14). This poor selectivity is due to their effect on the parasite apicoplast, an essential chloroplast-derived organelle that retains translation and DNA replication machinery of cyanobacterial origin. The lone validated exception is *Mycoplasma* removal agent (MRA), which contains an undisclosed fluoroquinolone and has successfully been used to clear *Mycoplasma* from cultures of apicomplexan parasites, including P. falciparum (6), Toxoplasma gondii, and Neospora caninum (15). The use of MRA has some downsides: clearance of *Mycoplasma* with MRA can take 1 to 3 weeks at the recommended concentration of 0.5 μg/mL, and some *Mycoplasma* strains have shown resistance to clearance with MRA (12, 13). Furthermore, MRA is relatively expensive at $696 per liter of culture medium.

Here, we report that the fluoroquinolone sparfloxacin is a safe, effective, and low-cost alternative to MRA for treatment of *Mycoplasma* contamination. Sparfloxacin cleared both MRA-sensitive and MRA-resistant *Mycoplasma* species from P. falciparum cultures at 1 and 4 μg/mL, respectively. Furthermore, we show that cultures of three different apicomplexan parasites can be maintained without deleterious effects on parasite growth at the concentrations of sparfloxacin required to clear *Mycoplasma*.

## RESULTS AND DISCUSSION

### Dose response of apicomplexan parasites to sparfloxacin.

**FIG 1:**
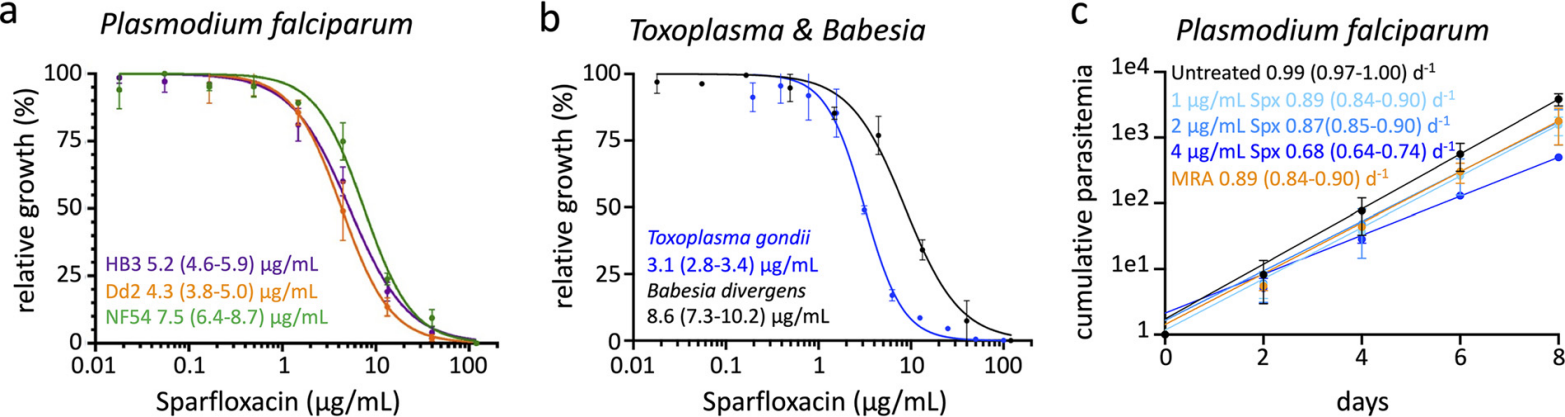
Dose-response curves of apicomplexan parasites to sparfloxacin. (a) Sparfloxacin dose-response curve of Plasmodium falciparum isolates of African (NF54; green), Southeast Asian (Dd2; orange), and South American (HB3; purple) origin based on 72-h SYBR green growth assays. The estimated 50% effective concentrations (EC_50_) with 95% confidence intervals are indicated based on *n* = 2 to 3. (b) Sparfloxacin dose-response curves of *B. divergens* isolate Rouen 1987 and T. gondii were measured using 72-h SYBR green and 96-h luciferase growth assays, respectively. The estimated EC_50_ values with 95% confidence intervals are indicated based on *n* = 2 to 3. (c) Representative growth curves of P. falciparum NF54 maintained at 1, 2, or 4 μg/mL sparfloxacin, MRA (0.5 μg/mL), or no drug. Growth rates with 95% confidence intervals are shown in day^-1^ based on *n* = 2.

To establish the maximal concentrations of sparfloxacin that cultures of apicomplexan parasites would tolerate, we measured the effect of sparfloxacin on the growth of three geographically diverse strains of Plasmodium falciparum (Fig. 1a), as well as Toxoplasma gondii and Babesia divergens (Fig. 1b), two other commonly cultured apicomplexan parasites. The 50% effective concentration (EC_50_) values of the tested isolates all fell within a narrow range, 3.1 to 8.6 μg/mL.

The effect of drugs inhibiting apicoplast replication does not manifest until parasites have invaded their next host cell, a phenomenon known as “delayed death” (16). Since the growth assays used for *Toxoplasma* and *Babesia* parasites covered multiple lytic cycles (Fig. 1b), these results already captured any delayed death effects of sparfloxacin on apicoplast replication. However, since P. falciparum divides by schizogony, and apicoplast replication only occurs every 48 h, our 72-h growth assays may not have fully captured any potential delayed death effects by sparfloxacin. To evaluate whether P. falciparum cultures exhibited any delayed death, we measured the effect of sparfloxacin over 4 replication cycles (Fig. 1c). The growth rates of the P. falciparum NF54 cultures were constant over 8 days at all concentrations of sparfloxacin, excluding the possibility of substantial delayed death effects, which would have resulted in a decrease of the growth rate after the first replication cycle. Compared to untreated cultures, there was a 10 to 12% decrease in the growth rate when parasites were maintained in the presence of 1 and 2 μg/mL sparfloxacin or 0.5 μg/mL MRA. Growth slowed by 31% at 4 μg/mL, but the cultures were maintained continuously for at least 3 weeks at this concentration (see Fig. 3b). While we did not quantify the effect on continuous growth, all the other P. falciparum strains, as well as the *Toxoplasma* and *Babesia* strains, were maintained at 4 μg/mL for prolonged periods.

### A low-cost, in-house PCR assay with internal controls is effective at detecting *Mycoplasma* contamination.

**FIG 2:**
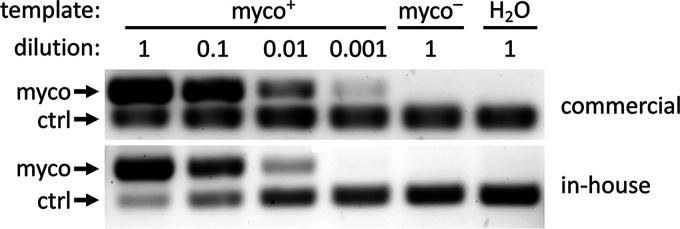
Detection of *mycoplasma* in P. falciparum cultures with commercial or in-house PCR assays. A commercial PCR assay was able to detect mycoplasma in the DNA of *mycoplasma*-positive (myco+) cultures even at 1:1,000 dilution, while the limit of detection for the in-house assay was between 1:100 and 1:1,000. The results from the undiluted *mycoplasma*-negative (myco^–^) and no template (H_2_O) controls are also shown. Images are representative of *n* = 2.

We compared the ability of an in-house PCR assay (based on universal *Mycoplasma* primers targeting 425 bp of the locus encoding the 16S rRNA gene [17]) to detect *Mycoplasma* in DNA isolated from cultures of P. falciparum to that of a commercially available *Mycoplasma* detection PCR kit. To provide an internal PCR control for negative *Mycoplasma* results, we included 100 fg of pUC18 plasmid and primers to amplify 316 bp of the plasmid backbone in the PCR master mix. The commercial PCR product was able to detect *Mycoplasma* in DNA extracted from *Mycoplasma*-positive cultures down to a 1:1,000 dilution at a per-sample cost of $6.15 (at the time of submission of this paper). The in-house PCR assay was also effective at detecting *Mycoplasma*, at a per-sample cost of $0.20, but the limit of detection was slightly lower at between 1:100 and 1:1,000 (Fig. 2). While the slightly lower sensitivity of the in-house assay suggests that further improvements are possible, it is still able to detect very low levels of contamination and its substantially lower cost makes it more amenable to routine use, even in resource-limited settings.

### Sparfloxacin is effective at clearing both MRA-sensitive and MRA-resistant *Mycoplasma* strains.

**FIG 3:**
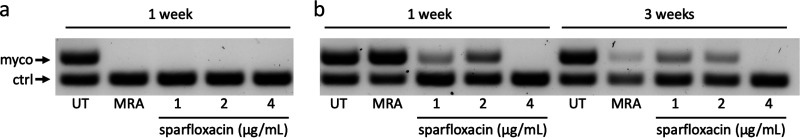
Effective clearance of *mycoplasma* with Sparfloxaxin. (a) Sparfloxacin reduced MRA-sensitive *mycoplasma* contamination in P. falciparum cultures below the level of detection of the in-house PCR assay after 1 week of treatment at all concentrations tested. (b) Sparfloxacin cleared MRA-resistant mycoplasma species from P. falciparum cultures after 1 week of treatment at 4 μg/mL, when MRA at the recommended 0.5 μg/mL failed even after 3 weeks of treatment. *Mycoplasma* was detected using the in-house PCR assay, and images are representative of *n* = 2. UT, untreated.

To evaluate the ability of sparfloxacin to clear *Mycoplasma*, mycoplasma-positive cultures were maintained either without treatment, with the manufacturer recommended 0.5 μg/mL of MRA, or with 1, 2, or 4 μg/mL of sparfloxacin. For most cultures, 1 week of treatment with MRA and all concentrations of sparfloxacin reduced *Mycoplasma* below the level of detection of the in-house PCR assay (Fig. 3a). However, *Mycoplasma* contamination in one culture proved to be resistant to clearance with MRA even after 3 weeks of treatment but was successfully cleared after 1 week of treatment with 4 μg/mL sparfloxacin (Fig. 3b).

Some fluoroquinolones, including sparfloxacin, can inhibit the mammalian HERG potassium channel. At higher concentrations than those used in this study, sparfloxacin was found to reduce the viability and growth of cell lines that expressed high levels of HERG (18), but sparfloxacin had no significant effect on growth of fibroblast cultures below 100 μg/ml (19).

This demonstrates that sparfloxacin can be used to treat even MRA-resistant *Mycoplasma* strains at concentrations that are well tolerated by a variety of apicomplexan parasites at a cost of $0.06 per liter of culture medium, making it a safe, effective, and low-cost alternative to MRA, with its cost of $696 per liter of culture medium.

## MATERIALS AND METHODS

### Parasite strains and maintenance.

Plasmodium falciparum strains NF54, Dd2, and HB3 and Babesia divergens strain Rouen 1987 (20) were maintained using standard malaria culturing techniques (21). Toxoplasma gondii strain RHΔ*ku80*::*NLuc* was maintained in human foreskin fibroblasts (HFF; ATCC SCRC-1041) using standard culturing techniques (22). Briefly, HFF/T. gondii co-cultures were maintained at 37°C with 5% CO_2_ in D10 medium (Dulbecco’s modified Eagle medium, 4.5 g/L glucose; VWR) supplemented with 10% Cosmic Calf serum (HyClone, GE Healthcare Life Sciences; SH30087.03), 10 mM HEPES, an additional 2 mM l-glutamine, and 10 mM Pen/Strep.

### *Mycoplasma* detection.

**FIG 4:**
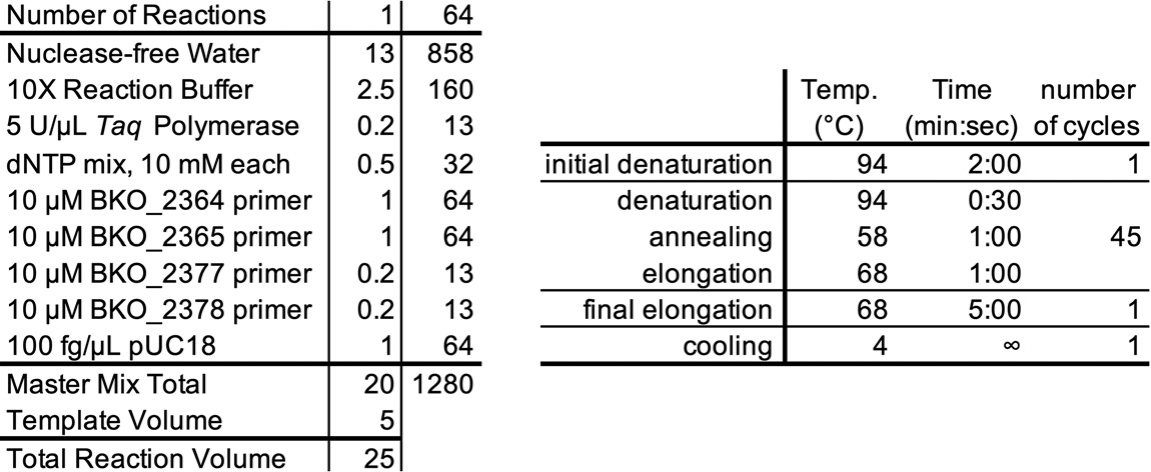
*Mycoplasma* detection PCR master mix composition (left) and cycling conditions (right). Indicated volumes are in microliters.

*Mycoplasma* contamination was detected using the e-Myco *Mycoplasma* detection kit (LiliF Diagnostics) according to the manufacturer’s instructions. Alternatively, *Mycoplasma* was detected using a custom PCR assay using the *Taq* polymerase manufacturer instructions and 0.4 μM of degenerate universal *Mycoplasma* detection primers (GTGGGGAGCAAAYAGGATTAGA/GGCATGATGATTTGACGTCRT) that target the 16S locus (17). As an internal control, each reaction also contained 100 fg of plasmid pUC18 and 0.08 μM primers (CCTGACGAGCATCACAAAAA/AGTCGTGTCTTACCGGGTTG) that amplify a 316-bp section of the plasmid backbone. The PCR master mix reagents (see Fig. 4) was comprised *Taq* polymerase and 10× reaction buffer (NEB; M0273S), a nucleotide mix containing 10 mM each dTTP, dATP, dGTP, and dCTP (NEB; N0446S), and 100 fg of the plasmid pUC18. pUC18 can be substituted with any other pBlueScript II-derived plasmid. Aliquots (20 μL) of the master mix were stored at −20°C until use. 5 μL of culture medium or 5 μL of DNA extractions (in TE) from cultures were added as template to bring the reaction volume to 25 μL.

### Dose-response assays.

Sparfloxacin (Thermo Scientific; J66358-09) was dissolved in dimethyl sulfoxide (DMSO) to a stock concentration of 2 mg/mL. For the asexual blood stages of P. falciparum and *B. divergens*, the dose response to sparfloxacin was determined using the 72-h SYBR green growth assay (23) as described in detail in reference 24. For T. gondii, 1,500 RHΔ*ku80*::*NLuc* tachyzoites were inoculated into each well of a 96-well plate containing confluent HFF cells, and the dose response was determined using a 96-h luciferase-based growth assay as described in detail in reference 22. In brief, the NanoLuc luciferase-expressing *Toxoplasma* parasites were allowed to invade human foreskin fibroblasts for 4 h. Noninvaded parasites were washed off, and the medium was replaced with fresh D10 supplemented with sparfloxacin. Ten concentrations generated by 2-fold serial dilution were tested starting at 100 μg/mL. After 96-h incubation, the medium was gently aspirated, and the infected host cells were extracted with lysis buffer (100 mM MES [4-morpholineethanesulfonic acid] [pH 6.0], 1 mM CDTA [*trans*-1,2-diaminocyclohexane-N,N,N′,N′-tetraacetic acid], 0.5% [vol/vol] Tergitol, 0.05% [vol/vol] Mazu DF 204, 150 mM KCl, 1 mM DTT [dithiothreitol], and 35 mM thiourea) containing 12.5 μM coelenterazine H and incubated at room temperature for 10 min prior to luminescence quantification using a BioTek Synergy H1 Hybrid plate reader. The luminescence signals from the sparfloxacin-treated cells were normalized against those from infected cells grown in plain D10 medium. The 50% inhibitory concentration (IC_50_) for each species was estimated by nonlinear fit using Prism (GraphPad).

### Effect of sparfloxacin on P. falciparum growth rate.

The growth of P. falciparum cultures was followed by measuring the culture parasitemia every other day for 8 days by flow cytometry after staining the live cultures with 16 μM Hoechst 33342 solution and 200 nM thiazole orange dye for 30 min at 37°C. Cultures above 2% were diluted to 0.5%, and the actual dilution factor was determined by measuring the parasitemia again after dilution using flow cytometry. The cumulative parasitemia was calculated by multiplying the percentage of infected cells by the overall dilution factor, and the growth rate was determined by exponential fit using Prism (GraphPad).

### Data availability.

No new materials were generated in this study.
